# A Rare Case of Isolated and Idiopathic Spontaneous Renal Artery Dissection in a Female Patient on Multiple Medications

**DOI:** 10.7759/cureus.5770

**Published:** 2019-09-26

**Authors:** Manjari R Regmi, Sarah-Grace A Carbrey, Priyanka Parajuli, Odalys Estefania Lara Garcia, Mukul Bhattarai

**Affiliations:** 1 Internal Medicine, Southern Illinois University School of Medicine, Springfield, USA; 2 Internal Medicine and Psychiatry, Southern Illinois University School of Medicine, Springfield, USA

**Keywords:** spontaneous renal artery dissection, poly-pharmacy, stimulant use, methylphenidate, dissection, dextroamphetamine

## Abstract

Constituting less than 25% of all renal artery dissections (RAD), isolated spontaneous renal artery dissection (SRAD) is a rare diagnosis that can cause subsequent renal infarction with impairment. The majority of SRAD cases are idiopathic. Management ranges from conservative, medical to endovascular, and surgical repair. We report a case of a young female on multiple medications who presented with SRAD. She presented with acute abdominal pain and was found to have isolated spontaneous renal artery dissection. The etiology of this patient’s isolated SRAD could possibly be pinned down to her multiple stimulant medications used after the major known causes were ruled out.

## Introduction

An isolated, spontaneous renal artery dissection (SRAD) is a dissection of the renal artery and is a very rare diagnosis. The first report of isolated, spontaneous renal artery dissection (SRAD) occurred in 1944 [1]. With the increasing use of imaging modalities and advanced endovascular technology, detection rates and incidence rates of RAD are on the rise. However, due to the limited number of cases of isolated RAD, there continues to be limited literature on other possible etiologies. Information on this subject is mostly limited to case series and individual case reports. The known associated conditions include malignant hypertension, atherosclerosis, connective tissue disorders (which include fibromuscular dysplasia, Marfan’s syndrome, and Ehlers-Danlos), blunt trauma, catheter procedures, and idiopathic causes. As mentioned by Jain et al., the leading cause of isolated SRAD is fibromuscular dysplasia, whereas, the second leading cause is still idiopathic contributing to 37% of SRAD [2]. As these cases are uncommon, it is difficult to reach the root cause of SRAD, especially when other signs and symptoms are not present.

## Case presentation

**Figure 1:**
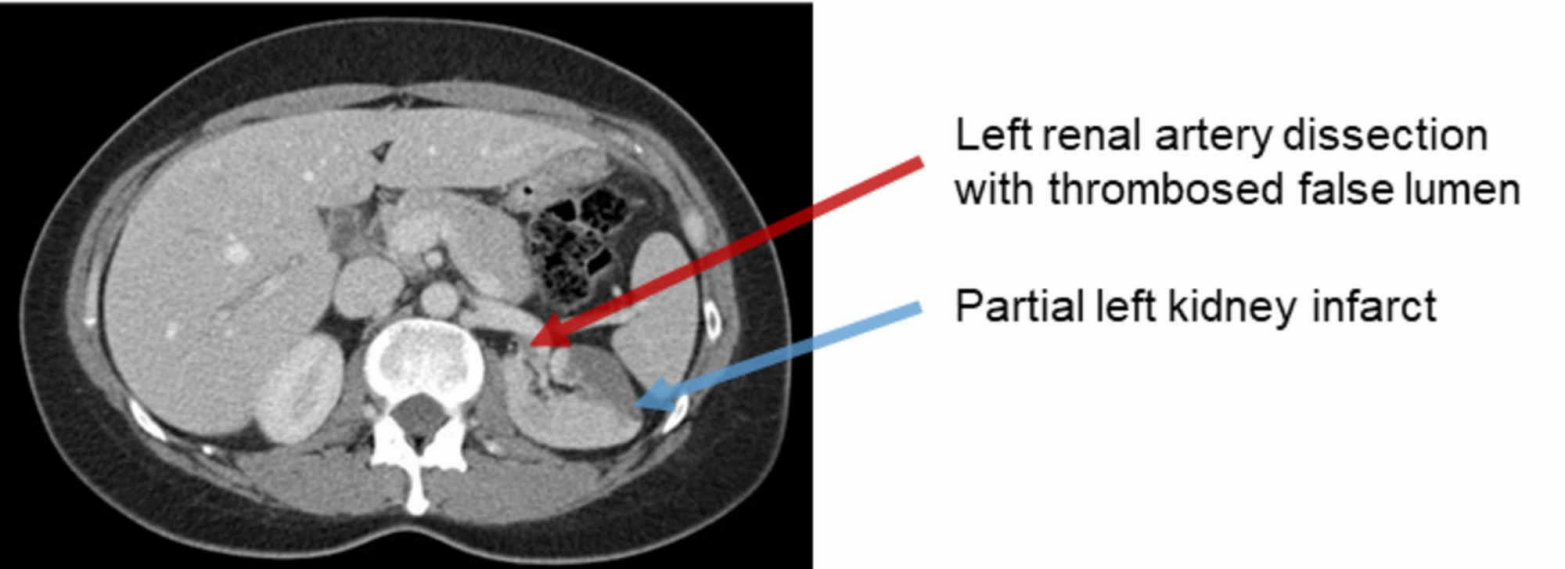
CT abdomen and pelvis: left renal artery dissection with a thrombosed false lumen and partial left kidney infarct CT, computed tomography

A 48-year-old female prison nurse (history of hypertension, migraine, major depressive disorder, and attention-deficit hyperactive disorder) presented after a sudden onset of severe sharp left lower quadrant abdominal pain and confusion. At that time, she was taking fluoxetine of 20 mg, hydrochlorothiazide of 12.5 mg, topiramate of 100 mg, eletriptan of 40 mg, amphetamine-dextroamphetamine of 30 mg, and methylphenidate of 10 mg. She reported increasing the frequency of her as-needed drug, methylphenidate, recently because of elevated stress at work. She had no personal or family history of vascular disease and connective tissue disorders. The physical exam was significant for mild tenderness in the left flank. Blood pressure (BP) was elevated at 156/97 on arrival with subsequent normalizations. Initial laboratory studies were within normal limits and urine toxicology was positive for amphetamines. Upon further evaluation, a CT scan of the abdomen and pelvis showed left renal artery dissection and left renal cortical infarct. Figure 1 shows the CT findings of the patient. The patient was given morphine for pain relief and her confusion resolved as her pain decreased. Within 48 hours of admission, repeated CT angiogram of chest, abdomen, and pelvis revealed unchanged left renal artery dissection with a thrombosed false lumen and partial infarction of the left kidney with no evidence of fibromuscular dysplasia. Further workup including CT head, ultrasound carotid arteries, and echocardiography was normal. Vascular surgery recommended conservative management and no surgical intervention. The pain was managed with morphine and Tylenol. Dextroamphetamine, methylphenidate, and eletriptan were held and BP remained controlled with hydrochlorothiazide. The patient was discharged on single antiplatelet therapy with a follow-up appointment with vascular surgery for repeat imaging.

## Discussion

The different types of RAD are as follows: 1) isolated (if localized to the renal artery without aortic involvement) or combined (if originating in the aorta with the involvement of renal artery) - static obstruction or dynamic obstruction; 2) primary (if associated with atherosclerosis or FM) or secondary (if associated with blunt trauma or endovascular interventions with wires/catheters); 3) idiopathic (all others without underlying pathology known or identified); unilateral or bilateral (one or both renal arteries involved); 4) spontaneous (SRAD; presenting in otherwise healthy individuals with acute presentation). Our patient herein had SRAD. 

With a male to female prevalence ratio of 4:1, SRAD is commonly found in healthy males in their 40s-60s. Due to the rarity of SRAD, information on a temporal cause-effect relationship is limited to case series and individual reports. Although there are various conditions associated with SRAD, the specific causal relationship and pathologies behind it are still not well understood. Some theories suggest an atherosclerotic alteration of vasa vasorum and its rupture as the underlying mechanism of SRAD [3].

The peculiar part of our patient was that she was on multiple medications, potentially interacting with each other, which have known sympathomimetic effects. The first drug, dextroamphetamine, an amphetamine salt, is known to exert catecholaminergic effects by stimulating the release of epinephrine and norepinephrine and also by inhibiting monoamine oxidase. Moreover, the second drug, eletriptan, class of abortive migraine drugs, are serotonin agonists on blood vessels and neurons and therefore, lead to vasoconstriction. The third drug, hydrochlorothiazide, and the fourth, topiramate, are urine alkalinizers that increase serum levels and decrease the excretion of dextroamphetamine. The fifth drug, fluoxetine increases serum levels of dextroamphetamine due to CYP2D6. In summary, two of the five drugs were responsible for sympathomimetic effects. The significant interactions of the other three drugs lead to an increased level of dextroamphetamine and therefore, amplified the catecholaminergic effects [4]. Amphetamine derivatives, combined with triptan can lead to intimal hypertrophy due to vasoconstriction or medial vasa-vasorum vascular changes that are highly associated with isolated RAD or RAD with aortic dissections. [5-6]

Amphetamine use leading to spontaneous coronary and aortic dissection has been reported in some cases; however, to the best of the authors’ knowledge, its association with isolated SRAD has not been reported yet. This case presentation provides strong evidence towards the validation of the theory that suggests an atherosclerotic alteration of vasa vasorum and its rupture as the underlying pathology behind SRAD. 

## Conclusions

SRAD is a rare but also a threatening condition that does not have a significant known temporal cause. While there are some cases reported of spontaneous coronary and aortic dissection in the setting of amphetamine use, there remains a paucity of published literature exploring this specific association. More research in this area is warranted in light of the rising rates of prescription stimulant use in the general population. Also, this case highlights that we have to be very cautious while using multiple, potentially interacting, medications. 
